# Comparison of Systemic Exposure Between Epinephrine Delivered via Metered-Dose Inhalation and Intramuscular Injection

**DOI:** 10.1089/jamp.2024.0025

**Published:** 2025-04-01

**Authors:** Jack Yongfeng Zhang, Mary Ziping Luo, Tony Marrs, Edward M. Kerwin, Don A. Bukstein

**Affiliations:** ^1^ Amphastar Pharmaceuticals, Inc., Rancho Cucamonga, California, USA.; ^2^ Clinical Research Institute and Allergy & Asthma Center, Medford, Oregon, USA.; ^3^ Allergy, Asthma, and Sinus Center, Milwaukee, Wisconsin, USA.

**Keywords:** albuterol, asthma, epinephrine, intramuscular injection, metered-dose inhaler, systemic exposure

## Abstract

**Background:**

Primatene^®^ MIST, an epinephrine metered-dose inhaler (MDI), has long been questioned by some medical professionals for asthma treatment despite having been approved by the Food and Drug Administration. One of the primary reasons for their concerns stemmed from potential cardiovascular complications following epinephrine administration. However, the majority of documented cardiovascular complications seemed to occur following the injection route of the epinephrine. The aim of this study was to evaluate the systemic exposure of epinephrine delivered through different administration routes and to understand its relationship with cardiovascular effects. Since albuterol inhalers are commonly recommended for asthma, albuterol was also studied as a comparator drug.

**Method:**

A randomized, evaluator-blinded, three-arm crossover study was conducted in 28 healthy adult subjects to compare the profiles of systemic exposure for epinephrine delivered by MDI versus epinephrine intramuscular (IM) injection and albuterol MDI. Serially sampled plasma epinephrine and albuterol levels were measured and compared between treatment groups. Safety was assessed by adverse events, serial vital signs, electrocardiograms (ECGs), and clinical laboratory tests obtained at each crossover dosing visit.

**Results:**

Systemic exogenous drug exposure for inhaled epinephrine MDI (39 pg/mL × hour) was ∼9 times lower than that of epinephrine IM (435 pg/mL × hour) and 122 times lower than that of albuterol MDI (3453 pg/mL × hour) after dose normalization. The C_max_ in epinephrine MDI (345 pg/mL) was approximately half of that of epinephrine IM (816 pg/mL) and that of albuterol MDI (681 pg/mL). Plasma drug concentrations for epinephrine MDI dropped rapidly to baseline (∼0.6 hour), while epinephrine IM took ∼8 hours, and albuterol MDI required more than 24 hours. Epinephrine MDI and albuterol MDI resulted in minimal, clinically insignificant changes in vital signs and ECGs, whereas epinephrine IM led to mild transient increases in systolic blood pressure, heart rate, and corrected QT interval.

**Conclusion:**

Epinephrine MDI (Primatene MIST) had ∼9 times lower systemic drug exposure (SDE) than that of epinephrine IM and ∼122 times lower than that of albuterol MDI. The lower SDE of inhaled epinephrine also correlated with reassuring safety findings, with no significant cardiovascular adverse effects found, compared with transient effects seen after IM epinephrine.

**Clinical trial registration number:**

NCT04207840.

## Introduction

Primatene^®^ MIST is the only Food and Drug Administration (FDA)-approved over-the-counter (OTC) epinephrine metered-dose inhaler (MDI) indicated for the temporary relief of mild symptoms of intermittent asthma in patients aged 12 years and older.^1^ The medication containing hydrofluoroalkane-134a (HFA) as the propellant was approved in November 2018 and replaced the reputable Primatene MIST CFC (chlorofluorocarbon), which was used safely as an OTC drug for over 50 years but was phased out due to environmental reasons associated with its CFC propellant.^2^

Despite showing positive safety and efficacy data and being approved by the FDA,^3^ the use of epinephrine inhalation products has long been questioned by some medical professionals.^4–6^ One of the primary reasons for their questions is the use of epinephrine as an active ingredient, which in high doses is associated with potential excessive cardiac stimulation. The association of epinephrine with cardiovascular effects is often tied to its nonselectivity to α- and β-adrenergic receptors. Epinephrine increases cardiac output by stimulating β1-adrenergic receptors within the heart and causes vasoconstriction by the activation of α1-adrenergic receptor.^7,8^ Therefore, the use of epinephrine is not recommended by the asthma guidelines except in the context of concomitant acute asthma and anaphylaxis or angioedema.^9–11^

For years, there have been reports regarding severe cardiovascular complications following epinephrine administration.^8,12–14^ The majority of these complications occurred when epinephrine was administered intravenously (IV).^13–14^ For example, a review paper assessing the safety of epinephrine found that a higher incidence of cardiovascular effects was observed for the IV route compared with other routes.^14^ These cardiovascular effects included arrhythmia, cardiac ischemia, stroke, angina, and hypertension.^8,13^ The findings led many to presume that the usage of epinephrine-related products could result in cardiac-related adverse effects. However, these are theoretical concerns with little if any supportive evidence for the safety or therapeutic concerns for epinephrine inhalation in treating asthma.^15^ In fact, during the 50-year history of Primatene MIST CFC, there was no substantial clinical evidence shown by any report or literature that serious cardiovascular effects occurred or were associated with the use of epinephrine inhalation. In contrast, studies have shown that few cardiovascular effects were seen following epinephrine inhalation.^16–18^ In one study, minimal cardiac effects were observed even after high doses of 10–30 inhalations of epinephrine, whereas subcutaneous injection of epinephrine caused a mild heart rate (HR) increase of <10 bpm immediately after injection.^17^ Furthermore, in another study comparing epinephrine MDI with albuterol MDI during nocturnal asthma, it was found that even after 14 actuations of epinephrine CFC MDI (220 μg per actuation), there were no increases in HR, while HR increases were observed in the albuterol MDI group.^16^

Since the binding of epinephrine to β1 receptors can occur over the surface of cardiac muscle cells,^19^ it is suggested that a high correlation may exist between the systemic exposure of epinephrine and cardiac-related outcomes. Therefore, the findings in the literature raise a question of whether the cardiac stimulation of epinephrine is attributed to the systemic bioavailability between different routes of administration of epinephrine.

The aim of this study is to evaluate the systemic exposure of epinephrine delivered through different routes of administration (inhalation vs. intramuscular [IM] injection) and determine whether dosing routes lead to differences in cardiovascular effects. IM epinephrine was selected as an area of interest since it has been used for acute severe or life-threatening asthma for decades.^11,20–22^ As albuterol inhalation has been recommended as an alternative when Primatene MIST CFC was phased out,^2^ albuterol MDI was also included as a treatment group and its systemic drug exposure (SDE) was assessed and compared. The pharmacokinetic (PK) profile and safety findings between epinephrine MDI (Primatene^®^ MIST, Armstrong), epinephrine injector (Epinephrine Injection Auto-injector, TEVA), and albuterol MDI (ProAir^®^ HFA, TEVA) are presented in this article.

## Materials and Methods

### Study design

This study was a randomized, safety evaluator-blind, single-dose, three-treatment arm, crossover study in healthy adult volunteers in the United States. The study consisted of a screening visit, three crossover study treatment visits, with treatments given in random order by a 3 × 3 Latin Square method, separated by an interval of 2–7 days, and a follow-up phone evaluation. During each treatment visit, subjects either self-administered epinephrine MDI (0.125 mg/inhalation, 2 inhalations) or albuterol MDI (0.09 mg/inhalation, 2 inhalations), or subjects received an epinephrine IM injection (0.3 mg epinephrine solution in 0.30 mL) (Table 1). For MDI inhalation, subjects were trained at screening and each study visit for correct dosing. For IM injection, subjects were instructed to lie down in a supine position and were given perpendicularly a single deep IM injection administered by study site staff into anterolateral aspect of the thigh (vastus lateralis site). Fasting (≥10 hours) was required prior to study drug dosing. Meals were served after the 4-hour post-dose PK sampling point and after the completion of end-of-study blood drawn.

**Table 1.** Study Treatment Summary

Product name	Epinephrine MDI (Primatene^®^ MIST)	Epinephrine IM auto-injector (generic of EpiPen^®^)	Albuterol MDI (ProAir^®^ HFA)
Treatment designation	Treatment A	Treatment B	Treatment C
Manufacturer	Armstrong	TEVA	TEVA
Product configurations	MDI	Auto-injector	MDI
Lot No.	PR05J8 (Exp. 09/2020)	9FM406 (Exp. 01/2021)	DAE00A (Exp. 11/2020)
Active ingredient	Epinephrine	Epinephrine	Albuterol Sulfate
Inactive ingredients	Ethanol (1%), hydrofluoroalkane (HFA 134a), polysorbate 80, thymol	Sodium chloride, sodium metabisulfite, sodium tartrate dihydrate, hydrochloric acid, water	Ethanol and hydrofluoroalkane (HFA 134a)
Dosage form	Microcrystalline suspension HFA MDI	Sterile solution	Microcrystalline suspension HFA MDI
Strength	0.125 mg/inhalation	0.3 mg/0.3 mL	0.09 mg/inhalation
Dose regimen	2 inhalations, 0.25 mg	IM Injection of 0.30 mg Epinephrine in 0.30 mL	2 inhalations, 0.180 mg
Route of administration	Oral inhalation	IM injection	Oral inhalation

IM, intramuscular; MDI, metered-dose inhaler.

The study was conducted in accordance with the current Good Clinical Practice, including the International Conference on Harmonization (ICH) Guidelines and the Declaration of Helsinki of the World Medical Association. The study protocol and informed consent form were approved by the IntegReview institutional review board, and written informed consent, which included compliance with the privacy provisions of the Health Insurance Portability and Accountability, was obtained from all subjects prior to initiation of the study. The study was registered on ClinicalTrials.gov (identifier NCT04207840).

### Study population

A sample of 24 subjects was planned for this study. The study population comprised healthy men and nonpregnant women between 18 and 50 years of age with a body mass of 18.0–30.0 kg/m^2^, who had normal resting blood pressure and HR. Subjects demonstrated negative alcohol/drug screen tests and negative HIV-Ab, hepatitis HBs-Ag, and HCV-Ab screen tests. Subjects were nonsmokers or had not used any tobacco products for at least 3 months prior to screening.

Subjects were ineligible if they had respiratory tract infection within 6 weeks before screening. Subjects were also excluded if they had clinically significant disease (i.e., cardiovascular, hematological, renal, neurological, hepatic, endocrine, psychiatric, or malignant diseases) that could impact the subjects and/or the study. Other exclusions included use of prohibited medications or intolerance to any of the study drug ingredients. Subjects could be withdrawn from the study early at the discretion of the investigator owing to medical safety, noncompliance, or administrative concerns.

### PK assessments

At each of the crossover study visits, 26 blood samples were collected from each subject at predose baseline (−30 to 0 minutes) and 1, 2, 3, 5, 7, 9, 12, 15, 18, 21, 25, 30, 40, 50, 60, 70, 80, 90 minutes and 2, 4, 6, 8, 12, 18, and 24 hours post-dose. Approximately 5 mL of blood was drawn at each sampling time point from a vein in the hand or arm via indwelling anticoagulated IV catheters or by serial venipuncture. Samples were collected in ice-chilled potassium—ethylenediaminetetraacetic sample tubes. The sample tubes were well mixed immediately and kept on ice or refrigerated. The blood samples were centrifuged at 4°C, 2000–3000 *g* for 20 minutes for plasma isolation. The isolated plasma from each sample was aliquoted to two duplicate 2 mL cryo vials, frozen on dry ice, and stored at less than or equal to −20°C until analysis. The samples were tested by a validated liquid chromatography/mass spectrometry/mass spectrometry method. Plasma epinephrine and albuterol were analyzed using Shimadzu Nexera X2 LC-30 UHPLC with triple quadrupole mass spectrometer Sciex QTRAP 6500 in multiple reaction monitoring mode and positive electrospray ionization. The chromatographic separation of the analytes employed an ACE Excel C18 100 × 2.1 mm column, with mobile phases comprising (1) 0.01% formic acid in water and (2) 0.01% formic acid in methanol, and a flow rate of 0.3 mL/min. The calibration range was 10–1000 pg/mL and a lower quantitative limit of 10 pg/mL or 10 parts per trillion (ppt).

The systemic exposure analysis was performed using per protocol population, which was defined as randomized subjects who completed all three crossover treatment visits on the study with evaluable PK data. From the data collected, plasma concentration–time curves were constructed for PK analysis. The systemic exposure of epinephrine MDI and epinephrine IM was evaluated based on:

1. the earliest PK time point (t_m_) when the plasma concentration of the epinephrine is reduced to levels of the same day baseline, which is defined as systemic exposure time;
2. the area under the curve of plasma drug concentration (AUC) for the total epinephrine (both exogenous and endogenous) from time 0 (predose) to t_m_, denoted as ${\mathrm{AUC}}_{0-t_{m}}^{\mathrm{TOT}}$;
3. the AUC for estimated exogenous epinephrine (estimated as the epinephrine level above the same day pre-dose baseline, i.e., baseline corrected) from time 0 to t_m_, which represents drug exposure (DE) of exogenous epinephrine and is denoted as ${\mathrm{AUC}}_{0-t_{m}}^{DE}$; and
4. the peak concentration from the PK curve (C_max_).

The study reports actual measured plasma concentration–time curves for PK analyses. For drug exposure ratios between treatment arms, dose-normalized C_max_ and AUC parameters were calculated to correct for varying administered doses (from 0.25 mg to 0.3 mg to 0.18 mg) in Treatments A (epinephrine MDI), B (epinephrine IM), and C (albuterol MDI). For example, when comparing drug exposures between epinephrine MDI and epinephrine IM, dose normalization was calculated as follows:

$${\left(\frac{B}{A}\right)}_{Q}=\frac{Q_{B}/d_{B}}{Q_{A}/d_{A}}$$

where Q is the PK parameter (i.e., C_max_ or AUC parameter) and $d_{A}$ and $d_{B}$ are doses for Treatments A and B, respectively.

Other parameters compared were the time corresponding to C_max_ (t_max_) and terminal elimination half-life (t_1/2_) of epinephrine. The mean and standard deviation of the study variables and the variances within and between subjects were estimated. Additional evaluation included relative bioavailability between epinephrine MDI and epinephrine IM. Similarly, the systemic exposure of albuterol MDI was also assessed using the same PK parameters as that of the epinephrine.

Systemic exposure of the study drugs was analyzed and compared using model-free based PK parameters, in which the PK parameters were obtained directly from the experimental data without using any model-based PK. The model-free PK analysis is efficient and robust such that it avoids the issue of suitability or potential biases associated with different PK models. These model-free PK parameters include AUC, C_max_, t_max_, and t_m_.

### Safety assessments

All adverse events (AEs) reported by subjects or observed by the study investigators were recorded. The incidence rate, severity (defined as events considered by the investigator to cause an inability to carry out activities), seriousness (defined as events that were fatal, hospitalization, disability, congenital abnormality, or other serious medical events), and type of AEs (i.e., possibly treatment-related) were assessed. Vital signs including systolic blood pressure (SBP), diastolic blood pressure (DBP), and HR, 12-lead ECG (routine and QT/QTc), serum glucose, and potassium were measured at baseline and at designated post-dose time points. Physical examinations and laboratory tests including a comprehensive blood count, fasting comprehensive metabolic panel, and urinalysis were evaluated at screening and end of study.

## Results

### Subject demographics

A total of 90 subjects were screened, and 28 subjects passed the screening, consented, and were randomized to receive study treatment. Study participants were 50% (*n* = 14) female and 50% (*n* = 14) male and a mean age of 37 years with the majority being Caucasian (46%) and African American (36%) (Table 2).

**Table 2.** Demographic Data

Subject characteristics	Crossover *n* = 28
Age (years)	
Mean ± SD	37.1 ± 7.6
Range	21–49
Gender, *N*(%)	
Male	14 (50.0%)
Female	14 (50.0%)
Race, *N*(%)	
Asian	1 (3.6%)
African American	10 (35.7%)
Caucasian	13 (46.4%)
Pacific Islander	0 (0.0%)
American Indian/Native	0 (0.0%)
Others	4 (14.3%)
Ethnicity, *N*(%)	
Hispanic or Latino	7 (25.0%)
Not Hispanic or Latino	21 (75.0%)
BMI	
Mean ± SD	25.5 ± 3.0
Range	18.4–30.0
Weight (kg)	
Mean ± SD	76.0 ± 13.7
Range	53.7–103.8
Height (cm)	
Mean ± SD	172.3 ± 10.3
Range	156.6–192.0

BMI, body mass index; SD, standard deviation.

**FIG. 1.**
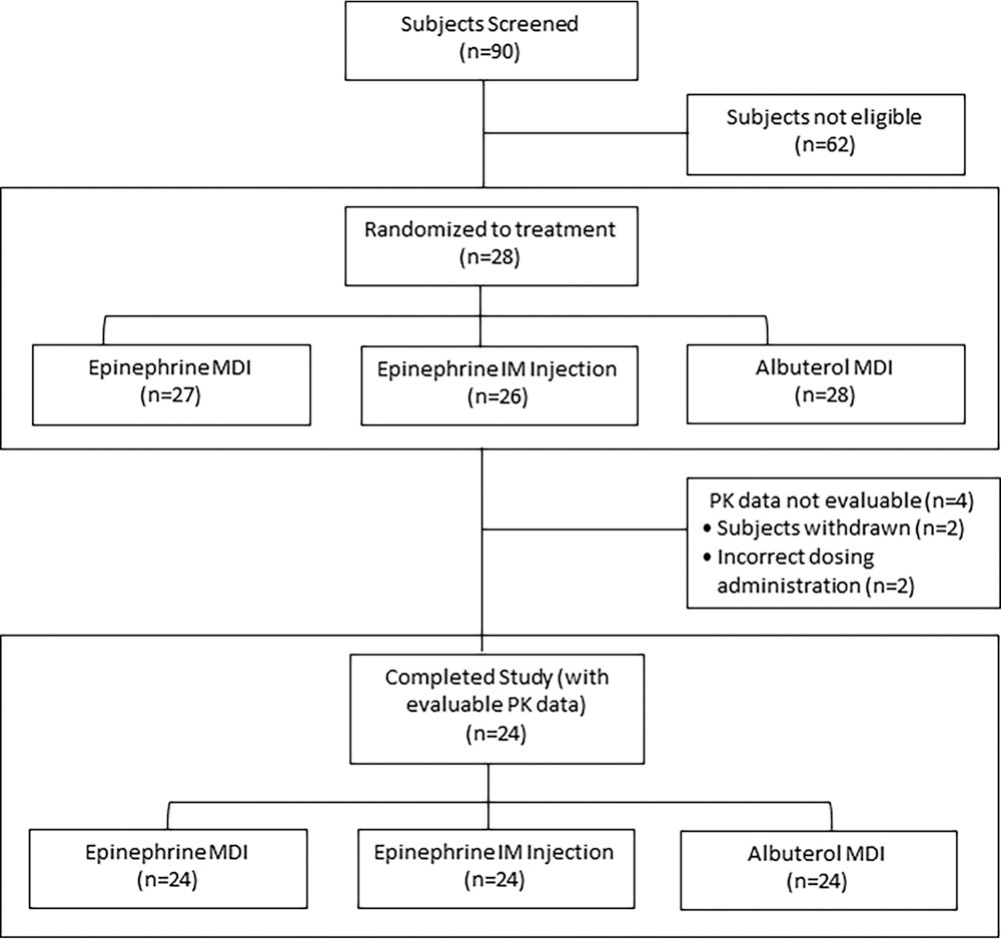
Subject disposition.

Among the randomized subjects, 27, 26, and 28 subjects received Treatment A (epinephrine MDI), Treatment B (epinephrine IM auto-injector), and Treatment C (albuterol MDI), respectively. Two subjects withdrew from the study, while data from two other subjects (one for epinephrine MDI and one for albuterol MDI) were not evaluable due to incorrect dose administration. Overall, 24 study subjects completed all three crossover dosing visits and were qualified with evaluable data for PK analysis (i.e., per-protocol population). Subject disposition is summarized in Figure 1.

### PK profiles

A total of 2106 PK samples were to be collected from the treated subjects. There were seven missing PK samples, which resulted in an overall of 2099 (or 99.7% of planned) PK samples collected for data analysis. The SDE data for the three-study drugs are provided in Table 3.

**Table 3.** Summary of Pharmacokinetic (PK) Parameters

	Treatment A	Treatment B	Treatment C	B/A^b^	C/A^b^
Study product					
Study drug	Epinephrine MDI	Epinephrine IM by auto-injector	Albuterol MDI	—	—
Dose, mg	0.25 mg	0.3 mg	0.18 mg	1.2	0.72
Delivery route	Oral inhalation	IM injection	Oral inhalation	—	—
Subject information					
Treated population (M, F)	27 (14, 13)	26 (14, 12)	28 (14, 14)	—	—
Per protocol population (PPP) (M, F)	24 (14, 10)	24 (14, 10)	24 (14, 10)	—	—
Age (year), for PPP	37.0 ± 6.9	37.0 ± 6.9	37.0 ± 6.9	—	—
Weight (kg), for PPP	77.5 ± 14.2	77.5 ± 14.2	77.5 ± 14.2	—	—
Height (cm), for PPP	174.0 ± 10.1	174.0 ± 10.1	174.0 ± 10.1	—	—
Systemic exposure time t_m_^a^					
t_m_, minutes	34 ± 51.4	460 ± 320	1440 ± 0	14	42
hours	0.57 ± 0.9	7.7 ± 5.3	24.0 ± 0.0		
Median (Min, Max), minutes	18 (3, 240)	360 (70, 1440)	1440 (1440, 1440)	20	80
Systemic drug exposure^a^					
AUC(0–t_m_) TOT, pg/mL × hour	55.6 ± 113	545 ± 196	3453 ± 854	8	86
AUC(0–t_m_) DE, pg/mL × hour	39.4 ± 70.4	435 ± 174	3453 ± 854	9	122
C_max_ and t_max_					
C_max_, pg/mL	345 ± 312.0	816 ± 444.0	681 ± 344.0	2	2.7
t_max_, minutes, Median (Min, Max)	2.0 (1, 80)	7.0 (1, 70)	12.0 (7, 360)	3.5	6
Half-Life, t_1/2_					
Half-life, t_1/2_, minutes	12 ± 26	148 ± 116	431 ± 99	12	35

^a^ t_m_—the time of the first PK point, after C_max_, when concentration is reduced to the same day baseline.

^b^ The data ratios of B/A and C/A for AUC and C_max_ are dose normalized. The other PK parameters are NOT.

TOT, total = exogenous + endogenous, which is the net exogenous contribution.

AUC, area under the curve; DE, drug exposure.

#### Systemic drug exposure of epinephrine inhalation versus epinephrine IM injection

**FIG. 2.**
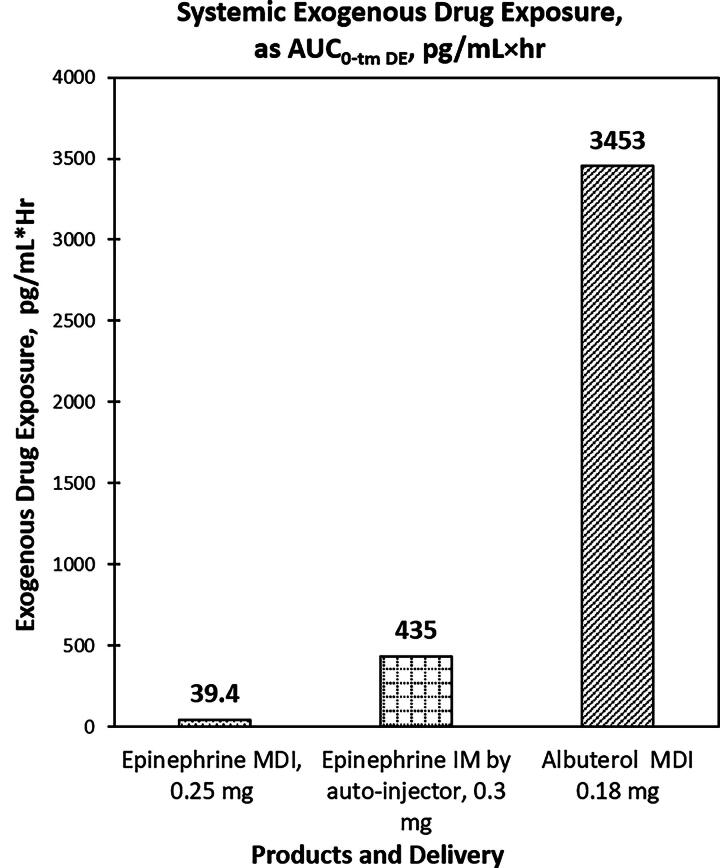
Systemic exogenous drug exposure for epinephrine MDI was approximately one degree of magnitude lower than that for epinephrine IM and was approximately two degrees of magnitude lower than that for albuterol MDI. IM, intramuscular; MDI, metered-dose inhaler.

**FIG. 3.**
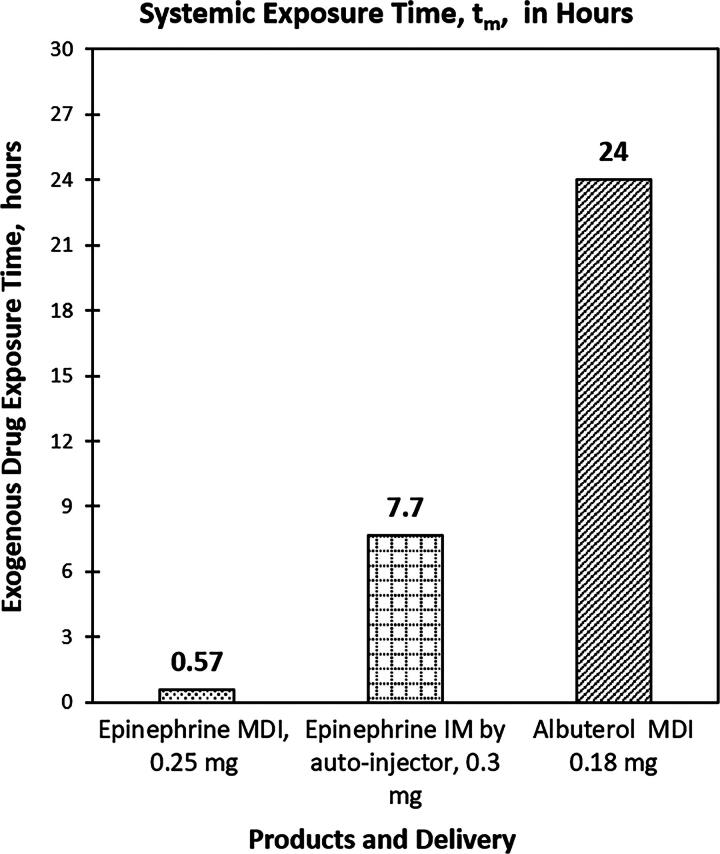
Systemic exogenous drug exposure time was lowest for epinephrine MDI.

The study results demonstrated that epinephrine by MDI inhalation had a lower SDE than that of epinephrine IM (Table 3 and Fig. 2). The systemic level of total epinephrine (exogenous and endogenous) was lower for epinephrine MDI (55.6 pg/mL × hour) compared with that for epinephrine IM (545 pg/mL × hour) as demonstrated by ${\mathrm{AUC}}_{0-t_{m}}^{\mathrm{TOT}}$. For estimated (baseline-corrected) exogenous epinephrine ( ${\mathrm{AUC}}_{0-t_{m}}^{DE}$), the SDE for epinephrine MDI (39.4 pg/mL × hour) was 9 times, or approximately one degree of magnitude, lower than that of epinephrine IM (435 pg/mL × hour) after dose normalization (Fig. 2). Furthermore, the plasma concentration of exogenous epinephrine through MDI inhalation dropped to baseline levels (t_m_) after 0.57 hours post-dosed (median of 18 minutes) compared with 7.7 hours (median of 360 minutes) from IM (Fig. 3).

Epinephrine MDI had a maximum drug concentration (C_max_) of 345 pg/mL, which was approximately half of the C_max_ for epinephrine IM (816 pg/mL). Moreover, epinephrine by MDI inhalation was absorbed more rapidly and also eliminated faster from serum compared with epinephrine IM by auto-injection. The time to maximum drug concentration (median t_max_) was 2 minutes for epinephrine MDI versus 7 minutes for epinephrine IM. Epinephrine MDI had a mean elimination half-life of 12.3 minutes, compared with the longer mean elimination half-life of 148.3 minutes of epinephrine IM (Table 3).

**FIG. 4.**
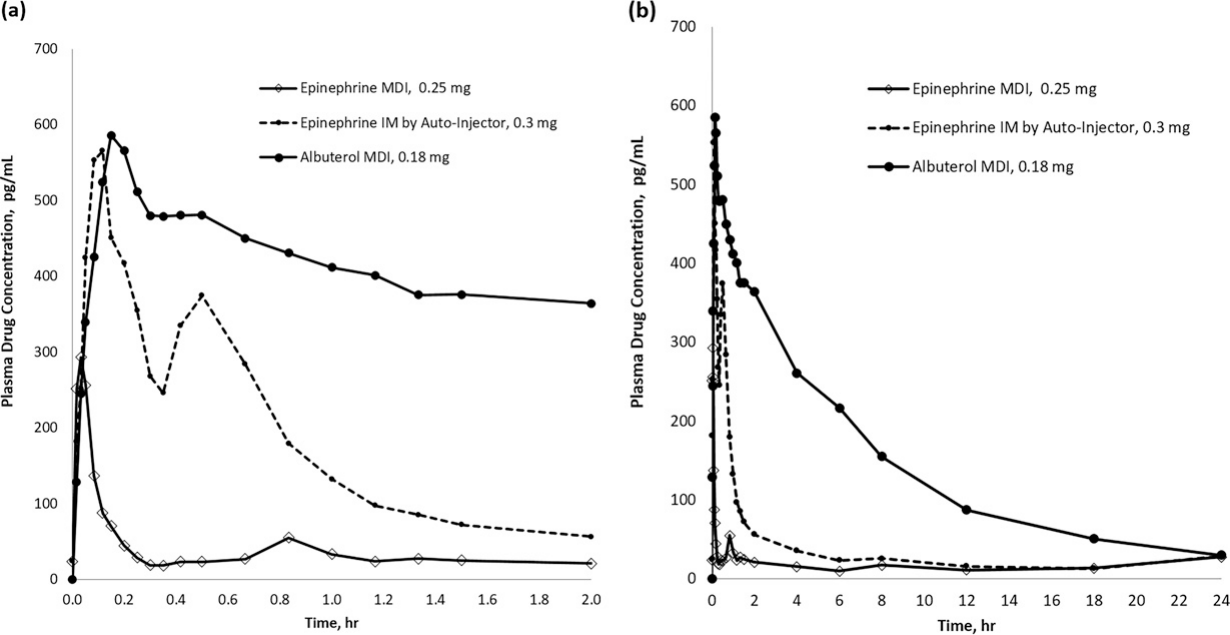
Plasma concentration–time curves of epinephrine MDI, epinephrine IM, and albuterol MDI **(a)** 0–2 hours and **(b)** 0–24 hours. The C_max_ for epinephrine MDI was approximately half that of the other two treatment groups, and the plasma drug concentrations returned to baseline levels after ∼0.6 hours compared with 8 and 24 hours in the epinephrine IM and albuterol MDI, respectively.

The bioavailability data between the epinephrine inhalation and IM products confirmed that only a small fraction of inhaled epinephrine (0.25 mg) was absorbed systemically compared with that of epinephrine IM (0.3 mg) (Fig. 4).

#### Systemic exposure of epinephrine inhalation versus albuterol inhalation

Inhalation of albuterol had a higher SDE of plasma albuterol at 3453 pg/mL × hour, which was calculated to be about 122 times or two degrees of magnitude higher than the SDE of plasma epinephrine of inhaled epinephrine after dose normalization (Fig. 2).

The maximum drug concentration (C_max_) of albuterol from albuterol inhalation was 681 pg/mL, which occurred at 12 minutes, whereas inhaled epinephrine reached maximum drug concentrations (345 pg/mL) at 2 minutes (Table 3). The mean elimination half-life of albuterol after albuterol MDI was 7.2 hours (about 431 minutes), and the mean elimination half-life of epinephrine after inhaled epinephrine was 12 minutes. Overall, albuterol inhalation resulted in a higher and more sustained SDE over 24 hours compared with epinephrine inhalation (Fig. 4).

### Safety assessment

A total of seven AEs were reported from six subjects throughout the course of the study. Among the seven AEs, four were from epinephrine MDI (three headaches and one nasopharyngitis), one from epinephrine IM (injection site pain), and two from albuterol MDI (nausea and headache). Only one AE (headache) from the albuterol MDI group was deemed to be possibly related to the study drug. The other six AEs were determined by the study investigator to be unrelated to the study drugs (Table 4) based on various factors including subject’s current medical history. All AEs were classified as mild and nonserious by the investigators, and all were resolved without residual effects.

**Table 4.** Summary of Adverse Events (AEs)

	All AEs reported		
	Treatment A	Treatment B	Treatment C
	Epinephrine MDI	Epinephrine IM auto-injector	Albuterol MDI
# of Subjects treated	27	26	28
# of subjects reported AEs	3 (11.1%)	1 (3.8%)	2 (7.1%)
# of AE occurrences	4 (14.8%)	1 (3.8%)	2 (7.1%)
AE per relationship to study drug			
Definitely not related	3 (11.1%)	1 (3.8%)	1 (3.6%)
Unlikely related	1 (3.7%)	0 (0.0%)	0 (0.0%)
Unknown	0 (0.0%)	0 (0.0%)	1 (3.6%)
Probably related	0 (0.0%)	0 (0.0%)	0 (0.0%)
Very likely related	0 (0.0%)	0 (0.0%)	0 (0.0%)

Epinephrine by IM auto-injector had an overall greater effect on vital signs and ECG compared with epinephrine by MDI (Table 5). The mean changes in SBP, DBP, and HR were minor for epinephrine MDI at 5 minutes to 24 hours post-dose, with changes in the range of 5–8 mmHg and 4–8 bpm, respectively. As for the epinephrine IM auto-injector, there was a mild transient increase in SBP of up to 12.69 mmHg at 5 to 15 minutes post-dose. A transient increase in HR of up to 13 bpm was also observed at 5 minutes post-dose but dropped to baseline levels after 1 hour. For ECG testing, minimal changes of 6–8 ms were observed in corrected QT interval (QTcF) after inhaled epinephrine. This change is considered negligible and well below the upper limit of 95% confidence interval of ΔQTcF versus baseline (95% confidence interval upper limit <10 ms). In contrast, the mean QTcF increased to 18 ms at 20 minutes post-dosed for epinephrine injection (Table 5).

**Table 5.** Summary of Vital Sign and Electrocardiogram (ECG) Results

Items				Δ vs. Baseline			Δ% vs. Baseline, %			Upper 95% CI for Δ		
Drug product	Epinephrine MDI	Epinephrine IM auto-injector	Albuterol MDI	Epinephrine MDI	Epinephrine IM auto-injector	Albuterol MDI	Epinephrine MDI	Epinephrine IM auto-injector	Albuterol MDI	Epinephrine MDI	Epinephrine IM auto-injector	Albuterol MDI
# of treated subject	27	26	28	27	26	28	27	26	28	27	26	28
SBP (mmHg), mean± SD												
Baseline	115 ± 12	114 ± 12	114 ± 12	—	—	—	—	—	—	—	—	—
05 minutes	118 ± 12	119 ± 12	117 ± 10	7.1 ± 5.8	12.7 ± 9.1	7.5 ± 6.5	6.1 ± 4.8	11.0 ± 7.6	6.8 ± 5.8	9.0	15.8	9.6
15 minutes	116 ± 11	120 ± 13	117 ± 11	7.2 ± 6.2	11.0 ± 9.5	7.6 ± 5.8	6.2 ± 5.2	9.7 ± 8.0	6.8 ± 5.4	9.2	14.2	9.5
60 minutes	111 ± 11	112 ± 10	115 ± 10	8.0 ± 7.6	9.0 ± 8.8	5.7 ± 4.1	6.9 ± 5.7	7.6 ± 6.4	5.1 ± 3.6	10.5	12.0	7.1
120 minutes	109 ± 11	110 ± 9	110 ± 10	8.6 ± 8.4	7.9 ± 8.4	6.3 ± 7.2	7.3 ± 6.3	6.6 ± 5.9	5.3 ± 5.6	11.4	10.7	8.6
24 hours or EOS	115 ± 10	115 ± 12	114 ± 11	7.3 ± 7.2	8.8 ± 7.7	8.4 ± 5.9	6.1 ± 5.5	7.6 ± 6.3	7.4 ± 5.5	9.7	11.4	10.3
DBP (mmHg), mean± SD												
Baseline	71 ± 8	69 ± 8	71 ± 9	—	—	—	—	—	—	—	—	—
05 minutes	70 ± 9	68 ± 8	72 ± 8	6.0 ± 3.5	5.9 ± 3.9	5.5 ± 3.7	8.6 ± 5.2	8.4 ± 5.6	7.7 ± 5.4	7.2	7.2	6.7
15 minutes	70 ± 9	66 ± 9	72 ± 8	6.0 ± 4.0	6.3 ± 5.5	5.9 ± 4.1	8.5 ± 5.7	9.0 ± 7.2	8.4 ± 5.7	7.3	8.2	7.2
60 minutes	69 ± 10	66 ± 8	68 ± 8	5.3 ± 4.7	6.6 ± 6.6	5.1 ± 4.0	7.6 ± 7.0	9.3 ± 9.2	7.1 ± 5.3	6.8	8.8	6.4
120 minutes	68 ± 8	68 ± 7	68 ± 8	6.0 ± 4.3	4.7 ± 5.7	5.0 ± 4.6	8.4 ± 5.8	6.6 ± 7.4	6.7 ± 5.8	7.4	6.6	6.4
24 hours or EOS	72 ± 8	71 ± 9	71 ± 7	5.7 ± 4.6	7.6 ± 6.0	5.3 ± 3.2	8.1 ± 6.6	11.0 ± 8.8	7.7 ± 5.0	7.2	9.6	6.3
HR (bpm), mean± SD												
Baseline	67 ± 12	69 ± 13	69 ± 10	—	—	—	—	—	—	—	—	—
05 minutes	71 ± 12	80 ± 14	72 ± 12	6.0 ± 4.6	13.2 ± 9.4	6.7 ± 4.4	9.1 ± 6.8	20.3 ± 15.8	9.6 ± 6.0	7.5	16.3	8.1
15 minutes	71 ± 11	74 ± 14	74 ± 12	6.7 ± 4.5	9.6 ± 7.0	8.4 ± 7.0	10.2 ± 7.0	14.2 ± 10.4	12.4 ± 11.4	8.2	12.1	10.7
60 minutes	66 ± 12	71 ± 13	68 ± 10	5.2 ± 4.0	9.5 ± 8.4	4.1 ± 3.1	7.7 ± 5.3	13.2 ± 10.1	5.9 ± 4.4	6.5	12.3	5.1
120 minutes	63 ± 10	66 ± 10	65 ± 9	5.6 ± 6.1	7.0 ± 8.2	7.2 ± 6.4	7.7 ± 7.3	9.7 ± 8.8	10.0 ± 7.8	7.6	9.8	9.3
24 hours or EOS	71 ± 11	71 ± 11	72 ± 11	7.9 ± 6.3	7.0 ± 6.5	7.0 ± 4.6	11.8 ± 9.5	9.8 ± 7.3	10.2 ± 6.7	9.9	9.2	8.5
ECG, QT (ms), mean± SD												
Baseline	405 ± 26	398 ± 24	401 ± 22	—	—	—	—	—	—	—	—	—
20 minutes	407 ± 24	402 ± 28	401 ± 22	8.3 ± 5.8	11.0 ± 10.0	7.9 ± 5.6	2.1 ± 1.4	2.7 ± 2.6	2.0 ± 1.4	10.2	13.8	9.7
120 minutes	412 ± 21	405 ± 24	408 ± 25	11.0 ± 9.0	11.7 ± 9.1	11.3 ± 11.8	2.8 ± 2.4	3.0 ± 2.5	2.8 ± 2.9	13.9	14.7	15.1
24 hours or EOS	396 ± 22	396 ± 22	395 ± 24	13.6 ± 12.6	12.3 ± 9.6	11.5 ± 7.2	3.3 ± 2.9	3.0 ± 2.3	2.9 ± 1.7	17.8	15.5	13.9
ECG, QTcF (ms), mean± SD												
Baseline	408 ± 14	404 ± 13	408 ± 13	—	—	—	—	—	—	—	—	—
20 minutes	409 ± 16	419 ± 21	409 ± 12	6.7 ± 4.9	18.0 ± 11.6	6.4 ± 5.2	1.6 ± 1.2	4.5 ± 2.9	1.6 ± 1.3	8.3	21.9	8.1
120 minutes	409 ± 13	409 ± 14	410 ± 17	6.8 ± 4.8	10.9 ± 6.9	8.8 ± 5.3	1.7 ± 1.2	2.7 ± 1.7	2.2 ± 1.3	8.4	13.2	10.5
24 hour or EOS	406 ± 12	403 ± 11	406 ± 14	6.2 ± 5.4	8.5 ± 5.7	8.9 ± 7.5	1.5 ± 1.3	2.1 ± 1.4	2.2 ± 1.8	8.0	10.4	11.3

CI, confidence interval; EOS, end-of-study; QTcF, corrected QT interval.

There were no clinically significant changes in potassium or glucose levels (Table 6). Only a small decrease (0.30 mmol/L) of serum potassium levels at 10 minutes post-dose was observed for epinephrine IM. For serum glucose, there was an increase of serum glucose levels of 17 mg/dL after IM auto-injector administration of epinephrine (Table 6).

**Table 6.** Summary of Serum Potassium and Glucose Results

Study treatment group	Treatment A	Treatment B	Treatment C
	Epinephrine MDI (*n* = 27)	Epinephrine IM auto-injector (*n* = 26)	Albuterol MDI (*n* = 28)
Plasma potassium (mmol/L), mean± SD			
Baseline	4.2 ± 0.3	4.2 ± 0.3	4.2 ± 0.3
10 minutes	4.1 ± 0.3	3.9 ± 0.3	4.1 ± 0.2
60 minutes	4.1 ± 0.3	4.0 ± 0.4	4.0 ± 0.3
24 hours	4.4 ± 0.4	4.4 ± 0.3	4.4 ± 0.3
Plasma glucose (mg/dL), mean± SD			
Baseline	83 ± 5	84 ± 5	83 ± 6
10 minutes	89 ± 6	97 ± 7	86 ± 6
60 minutes	84 ± 7	101 ± 16	83 ± 5
24 hours	83 ± 5	83 ± 5	83 ± 5

Albuterol MDI had similar post-dose vital signs (SBP, DBP, and HR), ECG, serum potassium, and glucose profiles to epinephrine MDI, where the changes were minor and not clinically significant, as demonstrated in Table 5 and Table 6. No clinically significant changes in CBC counts, metabolic blood tests, and urinalyses were observed for all treated subjects.

## Discussion

Despite epinephrine having been used for decades for the management of asthma,^11^ the use of epinephrine MDI often has been questioned by some organizations and committees due to a concern of potential excessive cardiac stimulation.^4–6,23^ The use of albuterol MDI is often recommended as an alternative.^6,24^ However, the concerns for epinephrine MDI do not distinguish between differences in SDE caused by different routes of administration of epinephrine, which could result in an entirely different outcomes. This article is the first to compare the SDE of epinephrine MDI oral inhalation versus epinephrine IM injection and albuterol MDI oral inhalation using a model-free PK analysis.

### Lower systemic drug exposure for epinephrine MDI

The study results demonstrated that only a small fraction of inhaled MDI epinephrine was distributed into the blood system in comparison with IM epinephrine or to systemic albuterol levels following inhaled albuterol. The estimated exogenous SDE ( ${\mathrm{AUC}}_{0-t_{m}}^{DE}$) for epinephrine by MDI inhalation (39 pg/mL × hour) is approximately one degree of magnitude lower than that for epinephrine by IM with auto-injection (435 pg/mL × hour) and two degrees of magnitude lower than that for albuterol MDI inhalation (3453 pg/mL × hour). This indicates that ∼90% of epinephrine delivered by MDI inhalation is not distributed into blood system, while much of the inhaled dose may be combined with β2-adrenergic receptors located in smooth muscle of bronchi to exert the asthma relief efficacy. In other words, only ∼10% of inhaled epinephrine entered the blood circulation, which resulted in a short systemic exposure time and a low SDE.

### Shorter systemic exposure time for epinephrine MDI

One of the main contributions to the lower SDE of epinephrine MDI is its short exposure time. Plasma levels of inhaled epinephrine rapidly reduced to baseline (the time is defined as “exposure time” and denoted as t_m_) by 18 minutes (median) post-dose, indicating a fast clearance time. In comparison, epinephrine IM had a drug systemic exposure time of 360 minutes (median), respectively, which is 20 times longer than the drug exposure times for epinephrine MDI. Besides AUC, C_max_, and t_max_, the exposure time (t_m_) is another model-free PK parameter for PK study of drugs with nonzero baseline.

### Comparison of Cmax and safety profile

The C_max_ for epinephrine MDI was approximately one-half of that for epinephrine IM. Moreover, inhaled epinephrine had no significant sympathomimetic effects, with no clinically meaningful changes in SBP, DBP, HR, or QT prolongation that would indicate any safety concern for epinephrine inhalation. As demonstrated in Table 5, epinephrine MDI and albuterol MDI caused clinically insignificant changes in HRs (∼6 bpm) and QTc-interval (∼7 ms). In contrast, epinephrine IM had more noticeable increases in HR (13 bpm) and prolonged QTc-interval (18 ms) during the early time points post-dose. The result of this single-dose study of epinephrine MDI in healthy volunteers is consistent with a previous long-term (6-month) multiple-dose study (*n* = 373) of epinephrine MDI in patients with mild-to-moderate persistent asthma, which found minimal and no clinically relevant changes in vital signs, ECG, serum glucose, and potassium.^25^ All AEs were nonserious and nonsignificant.

### Epinephrine MDI and its binding with β2-adrenergic receptors in the respiratory system

The findings in this study are consistent with previous literature where stimulation of β1 receptors in the heart muscles was found to be strongly correlated with systemic bioavailability of epinephrine.^18^ Although systemic IM or IV administered epinephrine binds to α- and β-adrenergic receptors nonselectively, inhaled epinephrine is delivered to the pulmonary airways that predominantly contain β2-adrenergic receptors in the bronchial smooth muscles.^18^ Therefore, epinephrine through inhalation has reduced effects on cardiac output, which is consistent with the observation of this study that the exogenous drug exposure for epinephrine by inhalation is approximately one degree of magnitude lower than that by IM injection. For example, a previous study showed minimal changes in cardiovascular effects despite using an ultrahigh dose (i.e., 10 inhalations) of inhaled epinephrine.^26^ Additionally, a separate long-term safety study found a low incidence of any cardiac-related AEs (e.g., HR increase and chest discomfort) during the 6-month duration of the study of using two puffs four-times-daily of inhaled epinephrine MDI.^25^ The 6-month safety study was also supported by more than 41,000 data points for vital signs and ECG and found minimal impacts from inhaled epinephrine in terms of SBP, DBP, HR, and QTc.^25^

When comparing with albuterol, the systemic epinephrine drug exposure was approximately two degrees of magnitude lower than the systemic albuterol drug exposure after albuterol inhalation. This suggests that distribution of epinephrine into blood system may be lower, and clearance may be more rapid, while the binding rate of epinephrine with β2-adrenergic receptors may also be faster than that of albuterol.

It may be worth noting that epinephrine and albuterol are different drugs with different molecular structures and molecular pharmacodynamics mechanisms. Therefore, the SDE of the two drugs is expected to be different. However, since both drugs are commonly used for asthma, it may still be relevant to study and compare their systemic effects.

### Strengths and limitations of this study

This study used a model-free PK analysis, where the parameters AUC, C_max_, t_max_, and t_m_ were obtained directly from the experimental data without relying on any PK models. This approach is generally preferred for studying SDE as it does not rely upon any assumptions about the underlying distribution of data, and it reduces any biases and variability associated with model-based approaches. While t_m_ is not as commonly used for PK analysis as other parameters such as AUC, C_max_ and t_max_, model-free t_m_ provides valuable information for assessing the duration of drug exposure and clearance time.

Although the study results found that a correlation exists between cardiovascular stimulation and systemic exposure of epinephrine, the study did not include IV routes of administration for epinephrine, which likely might have confirmed a higher incidence of cardiac stimulation from systemic exposures. Another potential limitation of this study is that it was a relatively small randomized trial. Therefore, a future study that includes IV routes of administration in a larger sample size may be needed to further validate the study results.

Furthermore, while the PK parameters such as C_max_ and AUC were dose normalized to account for the dosage differences between each product, other methods of normalization can also be performed such as target delivery (e.g., lung) for the MDI drugs. A future study directly comparing the dose delivered to the lung after epinephrine MDI, and albuterol MDI administration using target site normalization method may be considered.

Strengths of the present study include its crossover design, where 24 subjects were randomized and received all three drug doses sequentially, acting as their own controls. Subjects were carefully trained on inhalation technique and were observed during drug dosing. Additional strengths include intensive blood PK sampling time points (with 10 PK samples collected during the first 21 minutes after dosing), the high 99.7% completeness of PK samples analyzed, and the careful vital sign, ECG and blood chemistry monitoring, that was completed up to 24 hours after each dose. The use of an asthma active comparator adrenergic rescue inhaler, in the form of albuterol MDI, was another strength of our study.

Furthermore, the study included an adequate percentage of African American and Hispanic participants (Table 2), which reflects a representation of the target population in the real world^27^ associated with the use of inhaled epinephrine.

## Conclusion

Although the use of epinephrine MDI has long been questioned by some medical professionals due to the association of epinephrine with potential excessive cardiac stimulation, this study indicates that the cardiac effects of epinephrine are likely attributed to the systemic bioavailability in different routes of administration. When used according to labeled instructions, it was demonstrated that the SDE of epinephrine MDI (Primatene^®^ MIST) is approximately one degree of magnitude lower than epinephrine IM. The low SDE also correlated with reassuring safety findings, with no significant cardiovascular adverse effects found after dosing inhaled epinephrine, compared with transient effects seen after IM epinephrine. Furthermore, when compared with albuterol MDI, the SDE of epinephrine MDI was two degrees of magnitude lower than albuterol systemic exposures. The cardiac effects of epinephrine MDI and albuterol MDI are comparable and negligible when used as instructed in the labeling.

## Abbreviations Used

AE: adverse events
AUC: area under the curve
BMI: body mass index
bpm: beats per minute
CBC: complete blood count
CFC: chlorofluorocarbon
CI: confidence interval
C_max_: maximal concentration
DBP: diastolic blood pressure
DE: drug exposure
ECG: electrocardiogram
EOS: end-of-study
FDA: Food and Drug Administration
HFA: hydrofluoroalkane
HR: heart rate
ICH: International Conference on Harmonization
IM: intramuscular
IV: intravenously
MDI: metered-dose inhaler
OTC: over-the-counter
PK: pharmacokinetic
PPP: per protocol population
ppt: parts per trillion
QTc: corrected QT interval
SBP: systolic blood pressure
SD: standard deviation
SDE: systemic drug exposure
t_1/2_: elimination half-life
t_m_: systemic exposure time
t_max_: time for the maximal concentration
TOT: total (exogenous and endogenous epinephrine)
